# Values, Norms, and Peer Effects on Weight Status

**DOI:** 10.1155/2017/2849674

**Published:** 2017-02-28

**Authors:** Peng Nie, Wencke Gwozdz, Lucia Reisch, Alfonso Sousa-Poza

**Affiliations:** ^1^Institute for Health Care & Public Management, University of Hohenheim, 70599 Stuttgart, Germany; ^2^Department of Intercultural Communication and Management, Centre for Corporate Social Responsibility, Copenhagen Business School, Porcelænshaven 18a, 2000 Frederiksberg, Denmark

## Abstract

This study uses data from the European Social Survey in order to test the Prinstein-Dodge hypothesis that posits that peer effects may be larger in collectivistic than in individualistic societies. When defining individualism and collectivism at the country level, our results show that peer effects on obesity are indeed larger in collectivistic than in individualistic societies. However, when defining individualism and collectivism with individual values based on the Shalom Schwartz universal values theory, we find little support for this hypothesis.

## 1. Introduction

One of the many explanations that have been provided for the rising and persistent high prevalence of obesity in many countries is the changing societal values and norms associated with overweight [1, 2]. The reasoning is simple: as societies become more obese, individual attitudes towards being overweight change, with the acceptance of being overweight increasing and even ideal weight perceptions changing. Thus, both mean and desired body weights among adult Americans have increased from 1994 to 2002 [3]. In this sense, our weight perceptions and associated behaviours are being influenced by our (broadly defined) peers in society. Such peer effects can not only explain the persistence of obesity in society (it is difficult to change perceptions quickly) but also provide a good explanation for the rise in obesity (peers exert an externality which causes contagion).

There is a large body of literature that analyses the effects that peers have on body weight among children and adolescents [4–15] and adults as well [16–19]. Much of this literature analyses the effects that close friends have on body weight and behaviour [4, 6, 10, 14, 15, 17, 18]. Despite the methodological challenges in identifying peer effects, the general conclusion is that friends influence our physical activity [20], diets [13], and weight perceptions [2, 12]. A selection of studies uses a broader measure of peers, such as the average weight in a community or region [8, 11, 16]. As pointed out by Trogdon et al. [11], such broader measures of peers could capture norms and perceptions relating to an ideal body image. Blanchflower et al. [16] show that, for a given level of overweight, feelings of overweight increase the stronger an individual deviates from the average weight within a region. Maximova et al. [2], using data from the Quebec Child and Adolescent Health and Social Survey, demonstrate not only that a higher parental and schoolmate BMI is associated with greater misperception of weight status among children and adolescents but that overweight and obese youth are more prone to misperceive their own weight relative to nonoverweight peers.

Although it is widely accepted that peers affect individual's weight and associated behaviours, little is known about how this effect differs among countries, cultures, and societies. Prinstein and Dodge [21] have hypothesized that peer effects may be larger in collectivistic than in individualistic societies, stating that “it is possible that the effects of peers would be stronger for persons who are sensitive to the collectivistic orientation that may exist in their society [and] persons who believe that their culture is characterized by individualism may be more likely to be immune to the effects of peers.” There is virtually no research that tries to test this Prinstein-Dodge hypothesis. Although Mora and Gil [7] do not explicitly test this hypothesis, they do note that their peer effects among secondary school students appear to be larger in Spain than in the US and thus argue that “a more intense social life and stronger peer pressure in Mediterranean countries” may exist. Gwozdz et al. study [5] compares peer effects on obesity among children in several European countries. Using data from the IDEFICS study that covers several regions in eight European countries, they show that substantial variation in peer effects among the regions exists, with generally larger effects in the Spanish, Italian, and Cypriot regions than in the German, Swedish, Belgian, and Hungarian areas. Their tentative explanation for this observation is that peer pressure among children may be larger in more collectivist societies. This is the only study that tries to test the Prinstein-Dodge hypothesis and none has been conducted on adults. The reason for this dearth of research on this topic is that detailed cross-national data on obesity is necessary, which is rare.

In this study, we test the Prinstein-Dodge hypothesis using data from the European Social Survey (ESS). The advantage of using this data is that it not only covers many countries with different cultures but also has rich information on individual values. This latter point is particularly important as testing the Prinstein-Dodge hypothesis by simply comparing different countries ignores the fact that a significant amount of country-specific variation in values exists. Although Italy is generally classified as an individualistic society [22], Southern Italy is mostly collectivist. The ESS allows us to categorize* individuals* according to their values.

The remainder of the paper is structured as follows: Section 2 outlines the data and methods, Section 3 reports the results, and Section 4 concludes the paper.

## 2. Data and Methods

### 2.1. Data and Study Sample

The data for this analysis are taken from the European Social Survey (ESS), a biennial, academically driven, cross-sectional, pan-European social survey that documents and accounts for the interactions between Europe's changing institutions and the attitudes, beliefs, and behaviour patterns of its diverse populations [23]. Currently, the survey encompasses seven rounds (2002, 2004, 2006, 2008, 2010, 2012, and 2014) covering sociodemographics, media and social trust, politics, subjective well-being, and human values. Each round comprises randomly selected 1,000 to 2,000 interviews in each country [24]. One unique feature of the 2014 Round 7 dataset is its inclusion of a specific module of additional informative measures on the social factors of health and health inequalities. This Round 7 dataset covers 22 countries in Europe: Austria, Belgium, Czech Republic, Denmark, Estonia, Finland, France, Germany, Hungary, Ireland, Israel, Latvia, Lithuania, the Netherlands, Norway, Poland, Portugal, Slovenia, Spain, Sweden, Switzerland, and the UK.

Our analytic sample is restricted to those aged 15 and over for whom detailed information is available on demographics, household socioeconomics, and human values. Because the data on health measures—particularly body mass index (BMI)—are only available in Round 7, our final cross-sectional sample includes 37,917 observations in 21 countries. Note that Latvia takes part in Round 7 of ESS but data are not yet released.

### 2.2. Peers

In line with Blanchflower et al. study [16], we adopt a broad measure of peers at the country level. Specifically, we define peers as all individuals in the same country and in the same age band and gender, excluding the target individual *i*. We divide the individual age bands into 12 groups, <20, 20–24, 25–29, 30–34, 35–39, 40–44, 45–49, 50–54, 55–59, 60–64, 65–69, ≥70, and calculate the leave-out average BMI peer effects as follows:

(1) $$\begin{array}{c} {\overset{-}{BMI}}_{\left(i\right)gjc}=\frac{\left(\sum {BMI}_{igjc}-{BMI}_{igjc}\right)}{\left(N_{gjc}-1\right)}, \end{array}$$

where BMI_*igjc*_ represents individual *i*'s BMI in age band *j* and country *c* with the same gender *g*. ${\overset{-}{BMI}}_{(i)gjc}$ denotes the average BMI of peers in the same gender *g*, age band *j*, country *c* without individual *i*, and *N*_*gjc*_ designates the sample size of individuals in the same gender *g*, age band *j*, country *c*. Following Trogdon et al. study [11], we also adopt an alternative proxy that uses the proportion of overweight in the same gender *g*, age band *j*, and country *c* without individual *i*.

### 2.3. Dependent Variable

We employ self-reported BMI as a measure of individual bodyweight, which is calculated by dividing a person's weight in kilograms by the square of his or her height in meters (kg/m^2^). Such self-reports are prone to measurement error [24], yet in the absence of cross-national surveys that collect measured BMI, it is an unavoidable limitation. In our defense it should be noted that such data are widely used [16], and we have no reason to assume that systematic reporting biases may exist between countries or individuals with different values, which could confound our results.

As a robustness check, following Trogdon et al. study [11], we also employ a dummy of overweight equal to 1 if the respondent's BMI is 25 kg/m^2^ or above and 0 otherwise, which is based on the WHO criterion [25].

### 2.4. Individualism versus Collectivism

We define individualism and collectivism at both the country and individual level. Although defining a country as either collectivistic or individualistic is common in the literature [26, 27], simply comparing different countries ignores the fact that a significant amount of country-specific variation in values exists. The Hofstede country classification also has several drawbacks. First, Hofstede's categorization for comparing cultures is mainly based on the data from IBM employees among 53 nations or cultural regions and therefore his value dimensions distinguish different cultures among countries instead of individuals [28]. Second, most of the Hofstede items are related to work values, but they do not measure the range of human values associated with some life domains [28]. Third, this categorization is only based on Hofstede's theory post hoc without designing the questions of evaluating cultural values [29]. Fourth but not least, Hofstede's classification does not validate country-representation of data under analysis, thereby restricting its validity and reliability [29]. Thus, testing the Prinstein-Dodge hypothesis by characterizing* individuals* as collectivist or individualist is more convincing.

#### 2.4.1. Country-Level Characterization

At the country level, we apply the Individualism Score (IS) of Hofstede [22] (a higher score indicating a higher degree of individualism) to 21 countries in our sample and rank those countries in an ascending order, ranging from Portugal and Slovenia (IS = 27) to the UK (IS = 89). We then split those countries into two groups: individualistic (IS > 60) and collectivistic (IS ≤ 60). Based on this grouping, the individualistic countries are Finland, Germany, Switzerland, Norway, Ireland, France, Sweden, Denmark, Belgium, Hungary, Netherlands, and the UK. Collectivistic countries are Portugal, Slovenia, Spain, Israel, Austria, Czech Republic, Estonia, Lithuania, and Poland (a detailed illustration of Hofstede's IS by country in Table S2, in Supplementary Material available online at https://doi.org/10.1155/2017/2849674).

#### 2.4.2. Individual Level Characterization

The Israeli psychologist Shalom Schwartz has proposed a universal values theory based on the needs of individuals as biological organisms, requisites of coordinated social interaction, and survival and welfare of groups [30]. Schwartz (1994) defines ten values, namely, self-direction (SD, independent thinking, and action), stimulation (ST, novelty, and challenge in life), hedonism (HE, pleasure/gratification), achievement (AC, personal success), power (PO, social status, and prestige), security (SE, harmony, and stability of society), conformity (CO, self-restraint of actions), tradition (TR, acceptance of customs, and ideas), benevolence (BE, welfare enhancement), and universalism (UN, welfare tolerance, and protection). He further restructures these ten values into four dimensions: openness to change (SD, ST, and HE), self-transcendence (UN and BE), conservation (CO, TR, and SE), and self-enhancement (HE, AC, and PO). Particularly, self-transcendence such as UN and BE capture the values that highlight the importance of the welfare and interests of others, whereas self-enhancement such as PO and AC focuses on the pursuit of individual interests and relative success and dominance over others [31]. We calculate the centered score for each of the ten values (see Table S3) with the aid of the 21 human value items in the ESS. These 21 items are based on questions that ask respondents to assess the importance of certain values-related conditions. The 21 items are the following:

- Important to think new ideas and being creative
- Important to be rich, have money and expensive things
- Important that people are treated equally and have equal opportunities
- Important to show abilities and be admired
- Important to live in secure and safe surroundings
- Important to try new and different things in life
- Important to do what is told and follow rules
- Important to understand different people
- Important to be humble and modest, not draw attention
- Important to have a good time
- Important to make own decisions and be free
- Important to help people and care for others well-being
- Important to be successful and that people recognize achievements
- Important that government is strong and ensures safety
- Important to seek adventures and have an exciting life
- Important to behave properly
- Important to get respect from others
- Important to be loyal to friends and devote to people close
- Important to care for nature and environment
- Important to follow traditions and customs
- Important to seek fun and things that give pleasure

The responses are ranked on a 6-point scale from 1 = very much like me to 6 = not like me at all, and we then directionally rescale this variable to range from 1 = not like me at all to 6 = very much like me. Considering the aim of our study, we mainly focus on four values, namely, UN, BE, AC, and PO, because UN and BE are key collectivistic characteristics, whereas AC and PO are individualistic ones [31]. Based on the centered values of UN and BE, we create a dummy variable for collectivism that is equal to 1 if, for an individual, both UN and BE values are located at the upper quartile of each (UN and BE) distribution, and 0 otherwise. Similarly, we also create a dummy variable for individualism that is equal to 1 if both AC and PO values are in the upper 25% of each distribution, and 0 otherwise. As a robustness check, we also reset the cut-off point as 50% instead of 25%.

In Table S2, we also include the proportion of individualists and collectivists at the country level. It is interesting to note that there are some marked differences between the country-level and the individual-level classifications. Thus, in some countries that according to Hofstede [22] are classified as collectivistic, the proportion of individualists (calculated at the individual level) is in fact larger than the proportion of collectivists (and vice versa). For example, although Israel is according to Hofstede [22] classified as a collectivistic country, based on our individual-level classification, 13.1% of the population is individualistic, whereas only 6.3% is collectivistic. In the UK, which is a classic individualistic country according to Hofstede [22], 15.1% is classified as collectivistic, whereas only 5.5% is classified as individualistic. These results clearly highlight the shortcoming of using country-level classification schemes, as much individual heterogeneity is masked.

### 2.5. Explanatory Variables

Individual characteristics comprise four variables: gender, age groups, education, and marital status. Gender is a dummy equal to 1 if the respondent is male and 0 otherwise. As mentioned previously, age bands are categorized into 12 groups, <20, 20–24, 25–29, 30–34, 35–39, 40–44, 45–49, 50–54, 55–59, 60–64, 65–69, ≥70, and then recoded as a dummy with <20 as the reference age category. Education is measured on a 7-point scale of 1 = less than lower secondary, 2 = lower secondary, 3 = lower tier upper secondary, 4 = upper tier upper secondary, 5 = advanced vocational, 6 = lower tertiary, and 7 = higher tertiary and then recoded as a dummy with 1 = less than lower secondary as the reference group. Marital status is measured on a 5-point scale: 1 = never married, 2 = married, 3 = separated, 4 = divorced, and 5 = widowed. We recode it as dummies with never married as the reference group. We include relative household income, which is measured by the question: “Which of the descriptions on this card comes closest to how you feel about your household's income nowadays? 1 = very difficult on present income, 2 = difficult on present income, 3 = coping on present income, and 4 = living comfortably on present income.” We similarly convert it into a dummy with “very difficult on present income” as the reference group. Because some macroeconomic and demographic conditions are related to individual fatness [32–34], we also introduce three country-level characteristics: Gross Domestic Products (GDP) per capita, population density, and unemployment rate. Specifically, GDP per capita is calculated (at 2012 US dollars) as the aggregate of production divided by the population size. The population density (in 2011) is calculated as total population of each country divided by its land area. The unemployment rate is calculated for people aged 15–74 who are without work during the reference week, currently available for work, and actively seeking work.

### 2.6. Estimation Strategies

#### 2.6.1. Ordinary Least Squares (OLS)

To detect the existence of peer effects on individual BMI, we estimate the following OLS model:

(2) $$\begin{array}{c} {BMI}_{ic}=\beta_{0}+\beta_{1}P_{ic}+\beta_{2}I_{ic}+\beta_{3}H_{ic}+\beta_{4}C+\varepsilon_{ic}, \end{array}$$

where BMI_*ic*_ designates BMI of individual *i* in country* c* and *P*_*ic*_ represents the corresponding average BMI of the peers. *I*_*ic*_ is a vector of individual* i*'s characteristics, and *H*_*ic*_ denotes the household relative income dummies, *C* is a country dummy, *β*_1_ is the key coefficient of interest representing the association between peers and individual BMI, and *ε*_*ic*_ is the error term. We also investigate whether the average proportion of overweight among peers influences individual overweight by employing a probit model and use the same specification as in (2).

#### 2.6.2. Multilevel Mixed-Effects Generalized Linear Model (MMEGLM)

We then investigate the effects of both individual- and country-level characteristics on individual BMI by adopting the following multilevel mixed-effects generalized linear model:

(3) BMIic=Xicβ+Wcγ+μc+εic,i=1,…,Nc;  c=1,…,C,

where BMI_*ic*_ for individual *i* in country *c* is assumed to rely on observed and unobserved factors. *X*_*ic*_ denotes individual level characteristics such as age dummies, education, and marital status; *W*_*c*_ represents country-level characteristics such as GDP per capita, population density, and unemployment rate. And *ε*_*ic*_ and *μ*_*c*_ are unobserved individual effects and country effects, respectively, which both are assumed to be normally distributed and uncorrelated with *X*_*ic*_ and *W*_*c*_. *N*_*c*_ is the sample size of individuals within each country and* C* is the number of countries. *β* and *γ* are individual-level and country-level fixed effects, respectively. It is important to highlight that one of the substantial advantages of the MMEGLM technique is that a variety of different country-level fixed effects can be estimated by introducing country-level characteristics under analysis [35]. As Bryan and Jenkins [35] have emphasized, we may prefer to use OLS (with country-level fixed effects) if we are interested in the individual-level association with observed characteristics (*β*). Nonetheless, the MMEGLM approach could be a preferable option if the interest is in the effects of country-level fixed effects (*γ*) [35]. Thus, we cannot straightforwardly claim that MMEGLM is superior to OLS and the adaptation of those methods might largely rest on which factors are the focus of our interest.

#### 2.6.3. Quantile Regression

To examine whether mean peer BMI impacts different points of the individual BMI distribution differently (conditional on covariates), we estimate the following quantile regression model at the 25th, 50th, and 75th percentiles using the same specifications as in the OLS model:

(4) $$\begin{array}{c} {BMI}_{ic}^{q}=\beta_{1}^{q}P_{ic}+\beta_{2}^{q}I_{ic}+\beta_{3}^{q}H_{ic}+\beta_{4}^{q}C, \end{array}$$

where *q* denotes different quantile levels and *β*_1_^*q*^ is the key coefficient of interest.

## 3. Results

As Table S1 demonstrates, the mean BMI of individuals is 25.71, and the average peer BMI at the country level equals 25.70. The sample is predominately female (52.6%) and most are married (51.2%). Interestingly, 46.1% of respondents report that they are coping on present household income and 32.6% claim that they are living comfortably on present income. About 12% of individuals in our sample are classified as collectivists, whereas 9% are classified as individualists.

Results for the entire sample and all subsamples (collectivistic/individualistic) are reported in Table 1. The results in column 1, with only the mean peer BMI controlled for, show that the average peer BMI is significantly and positively associated with individual BMI (a coefficient equal to 0.905). In column 2, the peer effects remain significant and positive after introducing controls, but the magnitude is smaller (a coefficient equal to 0.367). In columns 3 and 4 it can be seen that peer effects are positively correlated with individual BMI, but the effect is significantly stronger in collectivistic regions than individualistic regions (0.423 versus 0.163). As the demarcation of collectivistic versus individualistic is quite arbitrary, we conduct two robustness tests. First, we develop groups based on the five highest and five lowest ranking countries using Hofstede's IS. Second, we also introduce the IS as a continuous variable into our model. When only using top 5 and bottom 5 countries (individualistic countries: Denmark, Belgium, Hungary, the Netherlands, and the UK; collectivistic countries: Portugal, Slovenia, Spain, Israel, and Austria), the results (Table S4) demonstrate that peer effects remain (a coefficient equal to 0.339; see column 1). Furthermore, for the split analysis of individualism versus collectivism, we only find the existence of peer effects in collectivistic countries (a coefficient equal to 0.300; see columns 2 and 3). When introducing the individualistic score and its interaction with average BMI, the results (Table S5) indicate that peer effects still exist when using the full sample (a coefficient equal to 0.566). We also note that the interaction effect is negative (albeit only marginally significant), indicating that the peer effect is stronger in collectivistic societies. Furthermore, when it comes to estimates from individual-based grouping, results reveal that peer effects do not differ significantly between the two groups. This also holds true when using the 50% and above observations as the cut-off points (see panel A in Table S6).

To investigate the heterogeneous response of average peer BMI on different points of individual BMI distribution, we also estimate quantile regressions (Table 2). A few findings to note are as follows: first, as panel A illustrates, average peer BMI is significantly positive on all points of the BMI distribution. Nevertheless, individuals in the upper part of the distribution (75th percentile: 0.495) are more sensitive to average peer BMI than those in the median and lower part of the distribution (50th percentile: 0.434; 25th percentile: 0.374). This is a common finding in the literature [8, 11]. Second, regarding country-level grouping (panel B), results from individualistic regions indicate that peer effects exist, although magnitudes do not differ across the distribution. Results in collectivistic regions demonstrate that peer effects are stronger at the upper end of the distribution (25th percentile: 0.374, 50th percentile: 0.513, and 75th percentile: 0.539; see panel C). Third, there is evidence that, based on the country-level specification, peer effects tend to be larger in collectivistic than in individualistic countries, especially at the upper end of the distribution. Finally, when using the individual-based grouping, results still confirm the existence of peer effects on all points the BMI distribution, yet magnitudes do not significantly differ between individualistic and collectivistic individuals (see panels D and E).

To check the potential effects of country-level factors on individual BMI, we also estimate a MMEGLM. As Table 3 shows, these coefficients remain uniformly significant and positive even after controlling for GDP per capita, population density, and unemployment rate. Stronger peer effects can be observed in collectivistic regions when using a country-level classification (columns 2 and 3). However, once again, no significant differences can be observed between individualistic and collectivistic individuals when using the individual level classification. We find that population density uniformly and negatively correlates with individual BMI (columns 1–3). This is in line with the negative associations between population density and individual BMI reported by Näyhä et al. [36] for the Northern Finland, Rundle et al. [37], and Zhao and Kaestner [34] for the US.

Following Trogdon et al. [11], we also examine whether average proportion of overweight among peers affects individual overweight (see Table 4). We find that peer effects are present and the magnitudes are stronger among collectivistic groups (columns 2–5), when using a country-level classification, yet no significant differences exist when using the individual level definition. This also holds true when using the 50% and above observations as the cut-off points (see panel B in Table S6).

## 4. Discussion 

The aim of this paper is to test the Prinstein-Dodge hypothesis, that is, whether stronger peer effects can be observed in more collectivist societies. Although some studies allude to this possibility [5, 7], none have systematically tried to assess it. Our paper employs a novel combination of macro (country-base) and micro (individual-based) approaches to measuring individualism and collectivism, thereby capturing peer heterogeneities in cultures, norms, and societies. Using data on more than 37,000 adults aged 15+ in 21 countries from the ESS Round 7 we can only provide limited support for the Prinstein-Dodge hypothesis: although the commonly-used country-level classification [22] for individualistic and collectivistic societies does support the Prinstein-Dodge hypothesis, an individual-level classification does not. As the country-level classification suffers from a few drawbacks and, above all, cannot capture the large heterogeneity of values within countries that is, even within countries commonly depicted as collectivistic (individualistic), a large portion of individuals are in fact individualistic (collectivistic), the individual-level classification provides a stronger and more convincing test of the Prinstein-Dodge hypothesis.

Of course, accurately testing such a hypothesis is challenging as one would ideally need panel data with rich information on (measured) overweight (for both peers and individuals) and information on values and norms and to cover several different cultures. Such data is not available, so our analysis must contend with certain data limitations. Although our data is rich in information on values and norms and covers several different countries, measures of (self-reported) BMI as well as the composition of the peer groups are not ideal, yet often used in the literature. Despite these data limitations, our results do at least question the notion that collectivistic individuals are more prone to be affected by the average BMI of same age and same-sex individuals within a country. Although this does not rule out the possibility that more narrowly defined peers (e.g., close friends) may have different effects on individuals with different values, our results do imply that even individualists are not immune to changing societal norms or ideal weight perceptions.

## 5. Conclusion

Our analysis based on data from the European Social Survey tests the Prinstein-Dodge hypothesis that posits that peer effects may be larger in collectivistic than in individualistic societies. In this paper, this hypothesis is tested in the context of obesity, and we provide only limited evidence for it. On the one hand, we do observe stronger peer effects among collectivistic societies when basing our definition along the traditional Hofstede (2001) country classification. On the other hand, when using individual level data to classify individuals along the Shalom Schwartz universal values theory, we provide limited evidence that collectivistic individuals are more affected by peers.

## Supplementary Material

Table S1 reports the descriptive statistics. Table S2 shows Individualism Score (IS) at the country level using Hofstede's classification and also the proportion of individualists and collectivists at the individual-level classification. Table S3 documents Schwartz's 10 human values for 21 individual value items in the European Social Survey. Table S4 reports the OLS results of average BMI on individual BMI using the five highest and five lowest ranking countries (based on Hofstede's IS). Table S5 demonstrates the OLS results of average BMI on individual BMI when introducing IS and its interaction with average BMI. Table S6 reveals OLS/probit results of average BMI/overweight on individual BMI/overweight.

## Figures and Tables

**Table 1:** OLS estimates of average BMI on individual BMI (individualistic versus collectivistic).

	All (without controls)	All (with controls)	Individualistic	Collectivistic	Individualistic	Collectivistic
			Country-based		Individual-based	
	(1)	(2)	(3)	(4)	(5)	(6)
Average BMI	0.905^*∗∗∗*^	0.367^*∗∗∗*^	0.163^*∗*^	0.423^*∗∗∗*^	0.621^*∗∗∗*^	0.519^*∗∗∗*^
	(0.023)	(0.054)	(0.087)	(0.079)	(0.199)	(0.133)
95% CI	[0.860,0.950]	[0.261,0.473]	[−0.009,0.334]	[0.269,0.577]	[0.232,1.011]	[0.257,0.781]

*N*	37917	37917	22172	15745	4605	5529
Adj. *R*^2^	0.106	0.130	0.112	0.182	0.162	0.119

The dependent variable is individual BMI. At the country level, individualistic countries are Belgium, Switzerland, Denmark, Germany, Finland, France, Hungary, Ireland, Netherlands, Norway, Sweden, and UK. Collectivistic countries are Austria, Czech Republic, Estonia, Spain, Israel, Lithuania, Poland, Portugal, and Slovenia. At the individual level, individualistic is for those individuals having 75% or above centered values of achievement and power. Collectivistic is for those individuals having 75% or above centered values of universalism and benevolence. Controls are average BMI (from its country *∗* age band *∗* gender cell), individual characteristics (dummies of age groups (with <20 as the reference group), gender, marital status, and education), household income (4-point scale, with 1 = very difficult on present income as the reference), and country dummies. Robust standard errors are in parentheses; 95% confidence intervals (CI) are in brackets. ^*∗*^*p* < 0.1, ^*∗∗*^*p* < 0.05, and ^*∗∗∗*^*p* < 0.01.

**Table 2:** Quantile estimates of average BMI on individual BMI (individualistic versus collectivistic).

Panel A: all	25%	50%	75%

Average BMI	0.374^*∗∗∗*^	0.434^*∗∗∗*^	0.495^*∗∗∗*^
	(0.044)	(0.051)	(0.069)
95% CI	[0.287,0.460]	[0.335,0.533]	[0.360,0.630]
*N*	37917	37917	37917
Pseudo *R*^2^	0.113	0.100	0.079

	Country-based		

Panel B: individualistic	25%	50%	75%

Average BMI	0.264^*∗∗∗*^	0.289^*∗∗∗*^	0.216^*∗∗∗*^
	(0.072)	(0.072)	(0.099)
95% CI	[0.124,0.405]	[0.148,0.430]	[0.023,0.410]
*N*	22172	22172	22172
Pseudo *R*^2^	0.103	0.089	0.070

Panel C: collectivistic	25%	50%	75%

Average BMI	0.374^*∗∗∗*^	0.513^*∗∗∗*^	0.539^*∗∗∗*^
	(0.069)	(0.079)	(0.106)
95% CI	[0.240,0.509]	[0.358,0.669]	[0.332,0.746]
*N*	15745	15745	15745
Pseudo *R*^2^	0.138	0.129	0.107

	Individual-based		

Panel D: individualistic	25%	50%	75%

Average BMI	0.257^*∗*^	0.372^*∗∗∗*^	0.603^*∗∗∗*^
	(0.133)	(0.121)	(0.182)
95% CI	[−0.005,0.518]	[0.134,0.609]	[0.247,0.960]
*N*	4605	4605	4605
Pseudo *R*^2^	0.148	0.145	0.119

Panel E: collectivistic	25%	50%	75%

Average BMI	0.343^*∗∗∗*^	0.560^*∗∗∗*^	0.568^*∗∗∗*^
	(0.088)	(0.122)	(0.172)
95% CI	[0.171,0.515]	[0.322,0.798]	[0.232,0.905]
*N*	5529	5529	5529
Pseudo *R*^2^	0.102	0.092	0.079

The dependent variable is individual BMI. At the country level, individualistic countries are Belgium, Switzerland, Denmark, Germany, Finland, France, Hungary, Ireland, Netherlands, Norway, Sweden, and UK. Collectivistic countries are Austria, Czech Republic, Estonia, Spain, Israel, Lithuania, Poland, Portugal, and Slovenia. At the individual level, individualistic is for those individuals having 75% or above centered values of achievement and power. Collectivistic is for those individuals having 75% or above centered values of universalism and benevolence. Controls are average BMI (from its country *∗* age band *∗* gender cell), individual characteristics (dummies of age groups (with <20 as the reference group), gender, marital status, education), household income (4-point scale, with 1 = very difficult on present income as the reference), and country dummies (for full sample, Germany as the reference country). Bootstrapped standard errors are in parentheses; 95% confidence intervals (CI) are in brackets. ^*∗*^*p* < 0.1, ^*∗∗*^*p* < 0.05, and ^*∗∗∗*^*p* < 0.01.

**Table 3:** MMEGLM estimates of average BMI on individual BMI (individualistic versus collectivistic, with GDP per capita, unemployment, and population density).

	All	Individualistic	Collectivistic	Individualistic	Collectivistic
		Country-based		Individual-based	
	(1)	(2)	(3)	(4)	(5)
Average BMI	0.561^*∗∗∗*^	0.318^*∗∗∗*^	0.599^*∗∗∗*^	0.549^*∗∗∗*^	0.468^*∗∗∗*^
	(0.076)	(0.027)	(0.072)	(0.083)	(0.121)
95% CI	[0.412,0.711]	[0.266,0.371]	[0.458,0.741]	[0.385,0.712]	[0.231,0.705]
Log(GDP per capita 2012)	−0.102	−0.481^*∗∗∗*^	−0.181	−0.111	0.041
	(0.086)	(0.066)	(0.128)	(0.126)	(0.175)
95% CI	[−0.271,0.067]	[−0.611, −0.352]	[−0.431,0.070]	[−0.358,0.136]	[−0.303,0.385]
Log(population density 2011)	−0.131^*∗∗∗*^	−0.214^*∗∗∗*^	−0.197^*∗∗∗*^	−0.112	−0.098
	(0.032)	(0.056)	(0.074)	(0.110)	(0.067)
95% CI	[−0.194, −0.067]	[−0.324, −0.104]	[−0.343, −0.052]	[−0.327,0.103]	[−0.229,0.033]
Unemployment rate 2012	−0.015^*∗∗*^	−0.073^*∗∗∗*^	−0.005	−0.018	−0.014^*∗*^
	(0.007)	(0.019)	(0.004)	(0.015)	(0.008)
95% CI	[−0.029, −0.002]	[−0.109, −0.036]	[−0.013,0.002]	[−0.048,0.012]	[−0.031,0.002]
Constant	11.772^*∗∗∗*^	21.934^*∗∗∗*^	11.646^*∗∗∗*^	11.567^*∗∗∗*^	12.121^*∗∗∗*^
	(2.444)	(0.696)	(2.559)	(2.758)	(4.486)
95% CI	[6.982,16.562]	[20.571,23.298]	[6.630,16.663]	[6.162,16.973]	[3.329,20.913]

*N*	33553	20132	13421	3937	4815

The dependent variable is individual BMI. At the country level, individualistic countries are Belgium, Switzerland, Denmark, Germany, Finland, France, Hungary, Ireland, Netherlands, Norway, Sweden, and UK. Collectivistic countries are Austria, Czech Republic, Estonia, Spain, Israel, Lithuania, Poland, Portugal, and Slovenia. At the individual level, individualistic is for those individuals having 75% or above centered values of achievement and power. Collectivistic is for those individuals having 75% or above centered values of universalism and benevolence. Controls are average BMI (from its country *∗* age band *∗* gender cell), individual characteristics (dummies of age groups (with <20 as the reference group), gender, marital status, and education), household income (4-point scale, with 1 = very difficult on present income as the reference), translog GDP per capita, translog population density, and unemployment rate. Robust standard errors are in parentheses; 95% confidence intervals (CI) are in brackets. ^*∗*^*p* < 0.1, ^*∗∗*^*p* < 0.05, and ^*∗∗∗*^*p* < 0.01.

**Table 4:** Probit estimates of percentage of overweight on individual overweight (individualistic versus collectivistic, marginal effects).

	All	Individualistic	Collectivistic	Individualistic	Collectivistic
		Country-based		Individual-based	
	(1)	(2)	(3)	(4)	(5)
Proportion of overweight	0.406^*∗∗∗*^	0.270^*∗∗∗*^	0.345^*∗∗∗*^	0.310^*∗*^	0.494^*∗∗∗*^
	(0.053)	(0.077)	(0.084)	(0.164)	(0.136)
95% CI	[0.303,0.510]	[0.119,0.421]	[0.180,0.510]	[−0.011,0.631]	[0.228,0.760]

*N*	37917	22172	15745	4605	5529
Pseudo *R*^2^	0.102	0.089	0.135	0.144	0.094

The dependent variable is individual overweight status (1 if BMI ≥ 25 kg/m^2^, 0 otherwise). At the country level, individualistic countries are Belgium, Switzerland, Denmark, Germany, Finland, France, Hungary, Ireland, Netherlands, Norway, Sweden, and UK. Collectivistic countries are Austria, Czech Republic, Estonia, Spain, Israel, Lithuania, Poland, Portugal, and Slovenia. At the individual level, individualistic is for those individuals having 75% or above centered values of achievement and power. Collectivistic is for those individuals having 75% or above centered values of universalism and benevolence. Controls are proportion of overweight (from its country *∗* age band *∗* gender cell), individual characteristics (dummies of age groups (with <20 as the reference group), gender, marital status, education), household income (4-point scale, with 1 = very difficult on present income as the reference), and country dummies. Robust standard errors are in parentheses; 95% confidence intervals (CI) are in brackets. Marginal effects are reported. ^*∗*^*p* < 0.1, ^*∗∗*^*p* < 0.05, and ^*∗∗∗*^*p* < 0.01.
