# Supplementary material for: Values, Norms, and Peer Effects on Weight Status
Source: J Obes. 2017 Feb 28;2017:2849674. doi: 10.1155/2017/2849674 (PMC5350312; doi:10.1155/2017/2849674)
Supplement: Supplementary file 1 — Table S1 reports the descriptive statistics. Table S2 shows Individualism Score (IS) at the country level using Hofstede's classification and also the proportion of individualists and collectivists at the individual-level classification. Table S3 documents Schwartz's 10 human values for 21 individual value items in the European Social Survey. Table S4 reports the OLS results of average BMI on individual BMI using the five highest and five lowest ranking countries (based on Hofstede's IS). Table S5 demonstrates the OLS results of average BMI on individual BMI when introducing IS and its interaction with average BMI. Table S6 reveals OLS/probit results of average BMI/overweight on individual BMI/overweight. [file 2849674.f1.pdf]

Supplementary Materials

TABLE S1: Descriptive statistics.

Variable	Obs.	Mean	Std. Dev.
Dependent variable			
BMI (kg/m^2)	37917	25.71	4.73
Independent variable			
Average BMI (kg/m^2)	37917	25.70	1.74
Individual-based individualism and collectivism			
Collectivism	36682	0.12	0.32
Individualism	36542	0.09	0.28
Gender	37917	0.47	0.50
Age groups	Frequency	Percentage	
Age group: <20 years	1975	5.21	
Age group: 20-24 years	2310	6.09	
Age group: 25-29 years	2528	6.67	
Age group: 30-34 years	2768	7.30	
Age group: 35-39 years	3088	8.14	
Age group: 40-44 years	3008	7.93	
Age group: 45-49 years	3232	8.52	
Age group: 50-54 years	3268	8.62	
Age group: 55-59 years	3340	8.81	
Age group: 60-64 years	3202	8.44	
Age group: 65-69 years	3167	8.35	
Age group: ≥70 years	6031	15.91	
Education levels			
Education: less than lower secondary	3769	9.94	
Education: lower secondary	6355	16.76	
Education: lower tier upper secondary	6896	18.19	
Education: upper tier upper secondary	6746	17.79	
Education: advanced vocational	5426	14.41	
Education: lower tertiary	4178	11.02	
Education: higher tertiary	4547	11.99	
Marital status			
Marital status: never married	11173	29.47	
Marital status: married	19404	51.17	
Marital status: separated	212	0.56	
Marital status: divorced	3873	10.21	
Marital status: widowed	3255	8.58	
Household relative income			
Household income: very difficult on present income	1981	5.22	
Household income: difficult on present income	6093	16.07	
Household income: coping on present income	17495	46.14	
Household income: living comfortably on present income	12348	32.57	
Country			
Austria	1724	4.55	
Belgium	1675	4.42	
Switzerland	1490	3.93	
Czech Republic	1877	4.95	
Germany	2925	7.71	
Denmark	1459	3.85	
Estonia	1993	5.26	

Spain	1808	4.77
Finland	2025	5.34
France	1866	4.92
The UK	2040	5.38
Hungary	1615	4.26
Ireland	2062	5.44
Israel	2324	6.13
Lithuania	2087	5.50
The Netherlands	1879	4.96
Norway	1398	3.69
Poland	1553	4.10
Portugal	1219	3.21
Sweden	1738	4.58
Slovenia	1160	3.06

Average BMI is the average BMI separated by gender, age bands and country. For education, marital status, age groups, household Income, and country, we report the number of observations (frequency) and the percentage in each category. Obs. = observations. Std. Dev. = standard deviation.
Source: the European Social Survey (ESS) 2014

TABLE S2: Individualism versus collectivism at country/individual levels.

	Country-based		Individual-based	
Country	Individualism score	Individualistic versus collectivistic	Individualistic	collectivistic
Portugal	27	collectivistic	0.076	0.093
Slovenia	27	collectivistic	0.069	0.032
Spain	51	collectivistic	0.032	0.202
Israel	54	collectivistic	0.131	0.063
Austria	55	collectivistic	0.093	0.095
Czech Republic	58	collectivistic	0.172	0.037
Estonia	60	collectivistic	0.089	0.114
Lithuania	60	collectivistic	0.270	0.026
Poland	60	collectivistic	0.106	0.075
Finland	63	individualistic	0.038	0.210
Germany	67	individualistic	0.049	0.185
Switzerland	68	individualistic	0.075	0.115
Norway	69	individualistic	0.072	0.126
Ireland	70	individualistic	0.088	0.088
France	71	individualistic	0.033	0.193
Sweden	71	individualistic	0.040	0.222
Denmark	74	individualistic	0.074	0.137
Belgium	75	individualistic	0.063	0.081
Hungary	80	individualistic	0.156	0.046
The Netherlands	80	individualistic	0.076	0.085
The UK	89	individualistic	0.055	0.151

Source: At the country level, individualism score is from Hofstede (2001). At the individual level, individualistic is for those individuals have 75% or above centered values of achievement and power. Collectivistic is for those individuals have 75% or above centered values of universalism and benevolence.

TABLE S3: Schwartz's ten human values for 21 individual value items.

Ten Human Values	Items in index of the ESS
Conformity (CO)	vii, xvi
Tradition (TR)	ix, xx
Benevolence (BE)	xii, xviii
Universalism (UN)	iii, viii, xix
Self-Direction (SD)	i, xi
Stimulation (ST)	vi, xv
Hedonism (HE)	x, xxi
Achievement (AC)	iv, xiii
Power (PO)	ii, xvii
Security (SE)	v, xiv

Source: Schwartz (2003).

TABLE S4 OLS estimates of average BMI on individual BMI (using top 5 and bottom 5 countries).

	All	Individualistic	Collectivistic
	(1)	(2)	(3)
Average BMI	0.339^{***}	-0.065	0.300^{***}
	(0.072)	(0.112)	(0.100)
95% CI	[0.199,0.480]	[-0.284,0.155]	[0.104,0.497]
N	16903	8668	8235
Adj. R^2	0.111	0.091	0.163

The dependent variable is individual BMI. Individualistic countries are Denmark, Belgium, Hungary, the Netherlands and the UK. Collectivistic countries are Portugal, Slovenia, Spain, Israel and Austria. Controls are average BMI (from its country*age band*gender cell), individual characteristics (dummies of age groups (with <20 as the reference group), gender, marital status, education), household income (4-point scale, with 1=very difficult on present income as the reference) and country dummies. Robust standard errors are in parentheses, 95% confidence intervals (CI) are in brackets. * p < 0.1, ** p < 0.05, *** p < 0.01.

TABLE S5 OLS estimates of average BMI on individual BMI.

	All
Average BMI	0.566^{***}
	(0.113)
95% CI	[0.344,0.787]
Individualistic score	0.080
	(0.049)
95% CI	[-0.016,0.176]
Average BMI X Individualistic score	-0.003^{*}
	(0.002)
95% CI	[-0.007,0.0003]
N	37917
Adj. R^2	0.131

The dependent variable is individual BMI. Controls are average BMI (from its country*age band*gender cell), individualistic score and its interaction with average BMI, individual characteristics (dummies of age groups (with <20 as the reference group), gender, marital status, education), household income (4-point scale, with 1=very difficult on present income as the reference) and country dummies. Robust standard errors are in parentheses, 95% confidence intervals (CI) are in brackets. * p < 0.1, ** p < 0.05, *** p < 0.01.

TABLE S6: OLS/Probit estimates of average BMI/overweight on individual BMI/overweight (individual-based individualistic versus collectivistic).

	OLS estimates	
Panel A: dependent variable is individual BMI	Individualistic	Collectivistic
Average BMI	0.376***	0.356***
	(0.099)	(0.093)
95% CI	[0.183,0.570]	[0.174,0.538]
N	12236	12661
Adj. R^2	0.167	0.110
	Probit estimates	
Panel B: dependent variable is individual overweight	Individualistic	Collectivistic
Proportion of overweight	0.388***	0.420***
	(0.096)	(0.089)
95% CI	[0.199,0.577]	[0.246,0.595]
N	12236	12661
Pseudo R^2	0.135	0.086

The dependent variable is individual BMI or individual overweight status (1 if BMI ≥25 kg/m^2, 0 otherwise). Individualistic is for those individuals have 50% or above centered values of achievement and power. Collectivistic is for those individuals have 50% or above centered values of universalism and benevolence. Controls are average BMI or proportion of overweight (from its country*age band*gender cell), and its interaction with four human values factors (universalism, benevolence, achievement and power), individual characteristics (dummies of age groups (with <20 as the reference group), gender, marital status, education), household income (4-point scale, with 1=very difficult on present income as the reference) and country dummies. Robust standard errors are in parentheses, 95% confidence intervals (CI) are in brackets. Marginal effects are reported in the panel B. * p < 0.1, ** p < 0.05, *** p < 0.01.
